# Fire frequency, as well as stress response and developmental gene control serotiny level variation in a widespread pioneer Mediterranean conifer, *Pinus halepensis*

**DOI:** 10.1002/ece3.9919

**Published:** 2023-03-21

**Authors:** Bastien Romero, Ivan Scotti, Bruno Fady, Anne Ganteaume

**Affiliations:** ^1^ INRAE, Aix Marseille Univ, RECOVER Aix‐en‐Provence France; ^2^ INRAE, URFM Avignon France

**Keywords:** cone serotiny, fire regime, genetic association, phenotypic plasticity, *Pinus halepensis*, single nucleotide polymorphisms

## Abstract

Many plants undergo adaptation to fire. Yet, as global change is increasing fire frequency worldwide, our understanding of the genetics of adaptation to fire is still limited. We studied the genetic basis of serotiny (the ability to disseminate seeds exclusively after fire) in the widespread, pioneer Mediterranean conifer *Pinus halepensis* Mill., by linking individual variation in serotiny presence and level to fire frequency and to genetic polymorphism in natural populations. After filtering steps, 885 single nucleotide polymorphisms (SNPs) out of 8000 SNPs used for genotyping were implemented to perform an in situ association study between genotypes and serotiny presence and level. To identify serotiny‐associated loci, we performed random forest analyses of the effect of SNPs on serotiny levels, while controlling for tree size, frequency of wildfires, and background environmental parameters. Serotiny showed a bimodal distribution, with serotinous trees more frequent in populations exposed to fire in their recent history. Twenty‐two SNPs found in genes involved in stress tolerance were associated with the presence‐absence of serotiny while 37 found in genes controlling for flowering were associated with continuous serotiny variation. This study shows the high potential of *P. halepensis* to adapt to changing fire regimes, benefiting from a large and flexible genetic basis of trait variation.

## Graphical abstract

While our results support a polygenic control of serotiny, they also show, for the first time, separate genetic bases for the presence/absence of serotinous cones (driven by genes encoding for stress tolerance and cuticle wax biosynthesis; this result is novel), on the one hand, and variation of serotiny level (driven by genes controlling flowering; this result is novel), on the other hand. This large and flexible genetic basis of trait variation may explain the high potential of *Pinus halepensis* for adaptation to changing fire regimes.

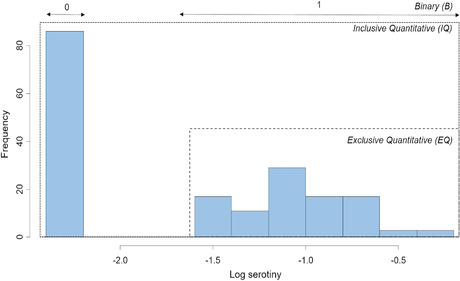

## 1 INTRODUCTION

Wildfires represent a highly selective disturbance for plant species (Bond & van Wilgen, 1996; Pausas & Keeley, 2009). In fire‐prone ecosystems, such as in the Mediterranean regions, several plant species have developed adaptations to fire. The specific types of adaptation depend on fire regime, which is defined, among other things, by fire frequency and intensity (Bradshaw et al., 2011; Keeley et al., 2011; Ne'eman et al., 2012). Three types of fire adaptive strategies have been reported in the literature (Pausas, 2015a). Among these, fire‐tolerant species survive a fire while fire‐embracer species base their strategy on postfire regeneration via resprouting (e.g., *Quercus* ilex L.) or via producing seedlings (e.g., *Pinus halepensis*). Fire‐tolerant species are adapted to fire regimes characterized by frequent low‐ to medium‐intensity surface fires (He et al., 2019; Murphy et al., 2013). These species (e.g., *Pinus sylvestris* L.) possess traits that mitigate fire impacts (e.g., thick bark, self‐pruning), enabling them to survive after fire (Fernandes et al., 2008; Keeley, 2012). By contrast, fire‐embracer species are adapted to fire regimes characterized by infrequent, high‐intensity crown fires leading to the death of the extant adult cohort (He et al., 2019). Fire‐embracer species that are obligate seeders present fire‐related traits enhancing crown flammability such as an aerated crown structure and dead fuel retention that create fuel continuity between the ground and the canopy, allowing the fire to spread vertically (Keeley, 2012; Ne'eman et al., 2004). This strategy releases propagules from competition for nutrients, water, and light by canopy vegetation during postfire regeneration.

The effects of fire regime on the phenotypic variation of traits are quite well studied (Engber & Varner, 2012; Enright et al., 2014; Pausas, 2015a, 2015b), but only a few studies found clear relationships between traits related to flammability and fire frequency (e.g., Pausas et al., 2012). Fire has shaped genetic diversity in species of fire‐prone environments (Fady, 2012) and within species and populations, the variability of fire‐related traits may be adaptive and response to changing fire regimes (Pausas, 2015a). The consequences of intra‐population heritable variation in fire adaptive traits on population fitness depend on the fire regime.

Among fire‐related traits, serotiny of pine cones presents a clear evolutionary response to changes in fire frequency (Keeley et al., 2011) and is evolutionary correlated with other fire‐related traits (Schwilk & Ackerly, 2001). Therefore, in this study, we used serotiny as an indicator for multi‐trait fire phenotypes in *Pinus halepensis* (see Budde et al., 2014 for *Pinus pinaster* Ait.). This functional trait is observed in fire‐embracer species and allows the plant to create a canopy seed bank, storing seeds in serotinous cones that can remain closed for several years until the occurrence of a fire event (He et al., 2012; Lamont et al., 1991; Pausas, 2015b). The heat shock induced by the high temperatures reached during a fire, for instance, triggers cone opening and seed release in a nutrient‐enriched environment with reduced competition. Therefore, serotiny increases fitness by increasing the success rate of seedlings after a fire (Causley et al., 2016; Hernández‐Serrano et al., 2013; Keeley et al., 2011; Pausas, 2015b).

The proportion of serotinous vs nonserotinous cones (i.e., serotiny level) can strongly vary among but also within species (Climent et al., 2014; Hernández‐Serrano et al., 2013; Peeler & Menges, 2018). An increase in serotiny level with fire frequency has been reported in many species. For instance, Ripa et al. (2020) found that serotiny level in *P. contorta* was higher in postfire recruited trees and could vary over a single generation after fire, suggesting a fast response to selection. The evolutionary response of serotiny to fire selective pressure rests on its variability among and within populations (Keeley et al., 2011; Lamont et al., 2020). Serotiny level varies widely among species, but several studies reported patterns in its distribution. For example, for maritime pine *Pinus pinaster*, the number of trees without serotinous cones was high compared with trees expressing serotiny (Budde et al., 2014; Castellanos et al., 2015). However, serotiny data observed by Budde et al. (2017) for the Aleppo pine (*Pinus halepensis*) was following a normal distribution.

Heritability of serotiny has been assessed in several studies concluding that serotiny could be considered as highly heritable with a simple genetic control (one locus with two alleles, i.e., Mendelian control) because changes appeared within solely a few generations (as observed in *Pinus contorta* and *Pinus banksiana*; Teich, 1970; Perry & Lotan, 1979; Wymore et al., 2011). With the improvement of the genetic tools and the apparition of association studies between genotype and phenotype, later studies showed that 11 to 17 loci explained 50% to 29% of the phenotypic variation of serotiny in *P. contorta* (Parchman et al., 2012) and in natural populations of *P. pinaster* (Budde et al., 2014), respectively, underlining a polygenic process driving serotiny. These results suggest that serotiny is controlled by more than one locus and demonstrate the feasibility of genomic association studies in natural populations. These studies also showed that association studies are very promising to assess the link between genotype and plant traits (De La Torre et al., 2019; Feduck et al., 2015; Pausas, 2015b).

Aleppo pine is a common and widespread fire‐embracer species of the central and western Mediterranean basin (Pausas, 2015a; Tapias et al., 2004; Wazen et al., 2020). In this species, serotiny level depends on fire regime, environmental conditions (e.g., soil nutrients, precipitations), and tree age, for instance (Cruz et al., 2019; He et al., 2012; Ne'eman et al., 2012). The identification of the genetic bases of serotiny in *P. halepensis* is important to assess the species' capacity to cope with expected future changes in fire regime (Dupuy et al., 2020). Castellanos et al. (2015), working on natural populations, found a heritability estimate of 10% while Hernández‐Serrano et al. (2014) found a higher value (20%) in a common garden experiment. Finally, Pinosio et al. (2014) identified 541 genes as differentially expressed between two natural trees sampled in populations undergoing different fire regimes, which may or may not be suggestive of genetic differentiation of those genes between the two populations.

These previous studies showed that serotiny in *Pinus halepensis* was genetically controlled and paved the way for genomic association studies. Considering that this trait is variably expressed among fire regimes, populations, and ontogenetic stages, it is important to run such association studies using samples that cover a wide span of conditions and therefore of potential serotiny values and/or sources of genetic variation. Given that it is difficult to disentangle the genetic basis of serotiny itself because loci associated with serotiny might be associated with other correlated fire adaptive traits, the goal of this study was therefore to identify a variety of candidate genes in *Pinus halepensis* that could underlie fire phenotypes. This work also aimed at understanding patterns of phenotypic variation in this trait when the fire history changes. To do so, we used an association approach in natural pine populations of southeastern France that vary in their fire history and in some environmental conditions.

## 2 MATERIALS AND METHODS

### 2.1 Study area and species

**FIGURE 1:**
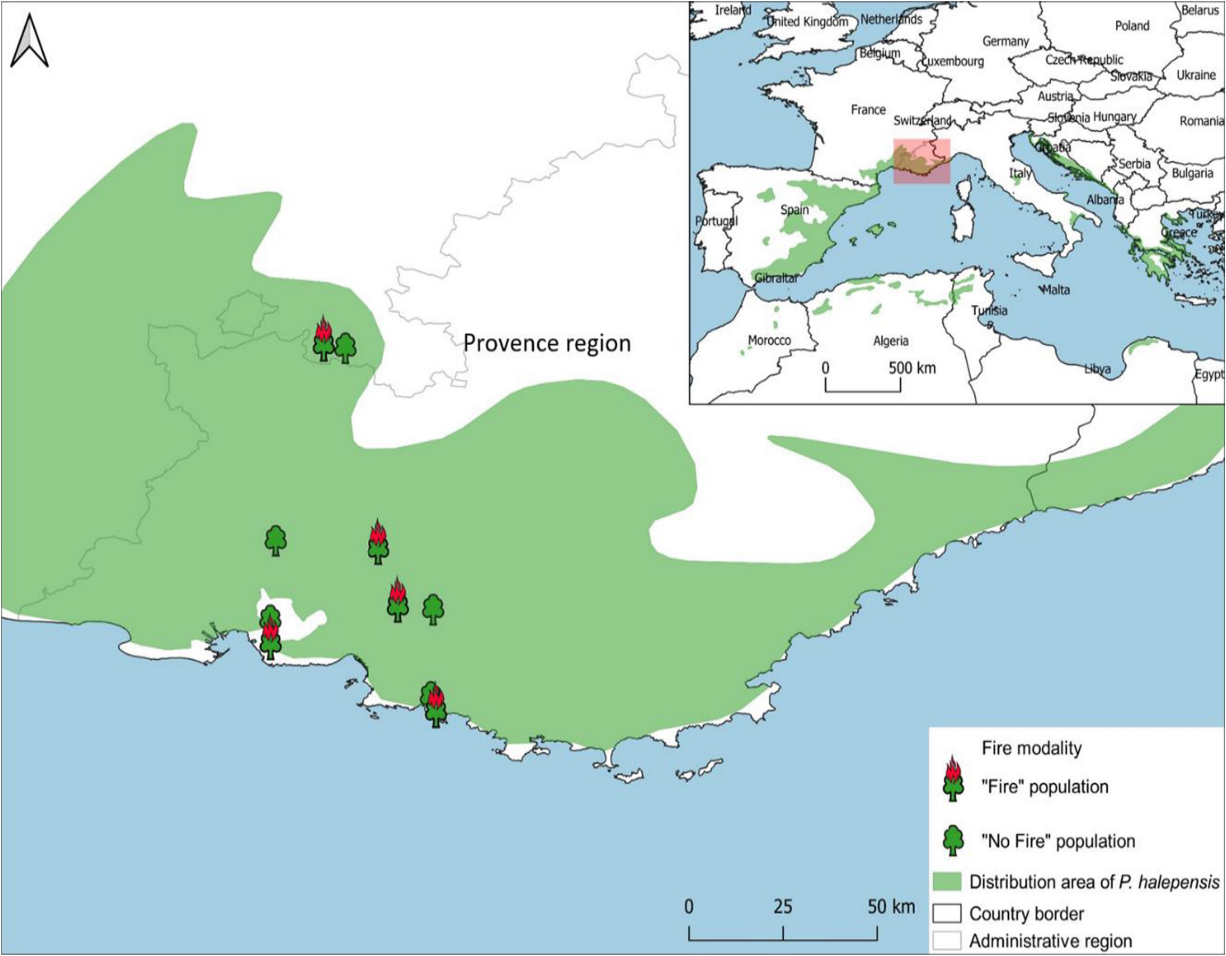
Map of the study area with “Fire” and “No‐Fire” sampling sites and *Pinus halepensis'* distribution area in green.

Sampling sites were located in southeastern France (Figure 1) and are characterized by a typical meso‐ to supra‐Mediterranean climate (Köppen, 1900; mild and humid winters, hot and dry summers), calcareous soils (i.e. limestone), and elevation ranging from 100 to 600 meters. Southeastern France is affected by moderate fire frequency compared with other countries of southern Europe (4657 fires and more than 21,000 ha burned during the 2010–2020 period, according to the regional fire database Prométhée; www.promethee.com), with a strong spatial variability. In the study area, as in other Mediterranean regions, fire has shaped most ecosystems but assessing the historical number of recurrent fires on a location is difficult due to a long fire history. Fire databases, when available, can provide reliable information on fire frequency during the last six decades, at most. In the current work, the recent fire history (60 years) was reconstructed using the regional fire database Prométhée (www.promethee.com) that has recorded fires in southeastern France since 1973 and the georeferenced fire perimeters recorded in the database of the Direction Départementale des Territoires et de la Mer (DDTM Bouches du Rhône) available from 1961. When the fire perimeters were not recorded in the database, we used satellite images and aerial photos to outline fire scars (e.g., delineation of unburned areas and fire boundary adjustment were performed using QGIS software).

The most common species in the study area are *Quercus ilex*, *Quercus pubescens*, and *Pinus halepensis* (Quézel, 2000). The latter species is an obligate seeder, bearing serotinous cones, and is one of the most studied Mediterranean trees (Ne'eman et al., 2004). *P. halepensis* is also a pioneer species adapted to drought, poor soil, high temperatures, and crown fire regimes (Pausas, 2015a).

### 2.2 Plant sampling and serotiny level measurement

Overall, 10 populations of *P. halepensis* were selected according to two fire modalities, half located in areas that were fire‐free from 1959 until 2018 (“No‐Fire” modality populations, PHNF) and the other half in areas having suffered at least one fire (“Fire” modality populations, PHF) during the same time period. The pairs “Fire”–“No‐Fire” were located between 5 and 25 km apart in order to minimize genetic divergence caused solely by drift (Lotterhos & Whitlock, 2015). The populations were sampled in sites where the past land use was the same in order to mitigate the impact of soil conditions on trait variation (See Romero & Ganteaume, 2020). In the current work, the sampling occurred in recent forests (as opposed to ancient forests that could have already been mapped in the 18th–19th century), mostly resulting from land abandonment that can provide adequate conditions for regeneration in the absence of fire. In each population, 19–20 mature and dominant trees, between 15‐ and 30‐years‐old (on average 22.03 ± 3.1‐years‐old), were sampled. In areas undergoing fires during the past 60 years, we only sampled populations when at least 10 years had elapsed since the last fire event in order to be sure of the sexual maturity of the trees that have grown postfire (Santos del Blanco et al., 2010). Furthermore, in order to reduce environmental differences between populations, we selected sample sites with as homogeneous environmental conditions as possible (slope, exposure, elevation, and past land use; see Table S1). However, Romero and Ganteaume (2020) found a significant effect of these factors on trait variation, working on the same database as the one used in the current study. This effect has therefore to be accounted for in the analyses (see below).

Serotiny measurements were carried out following Budde et al. (2014). Using binoculars, we counted, on each tree, 20 mature cones (older than 3 years) on healthy branches, avoiding dominated trees and those with a diameter smaller than 10 cm. Measurements were performed during the summer (June–July 2018), at least 48 h after a rain event when occurred to avoid counting cones closed due to high relative humidity. Tree serotiny level was calculated as the number of closed cones divided by the total number of cones (on 192 trees).

**FIGURE 2:**
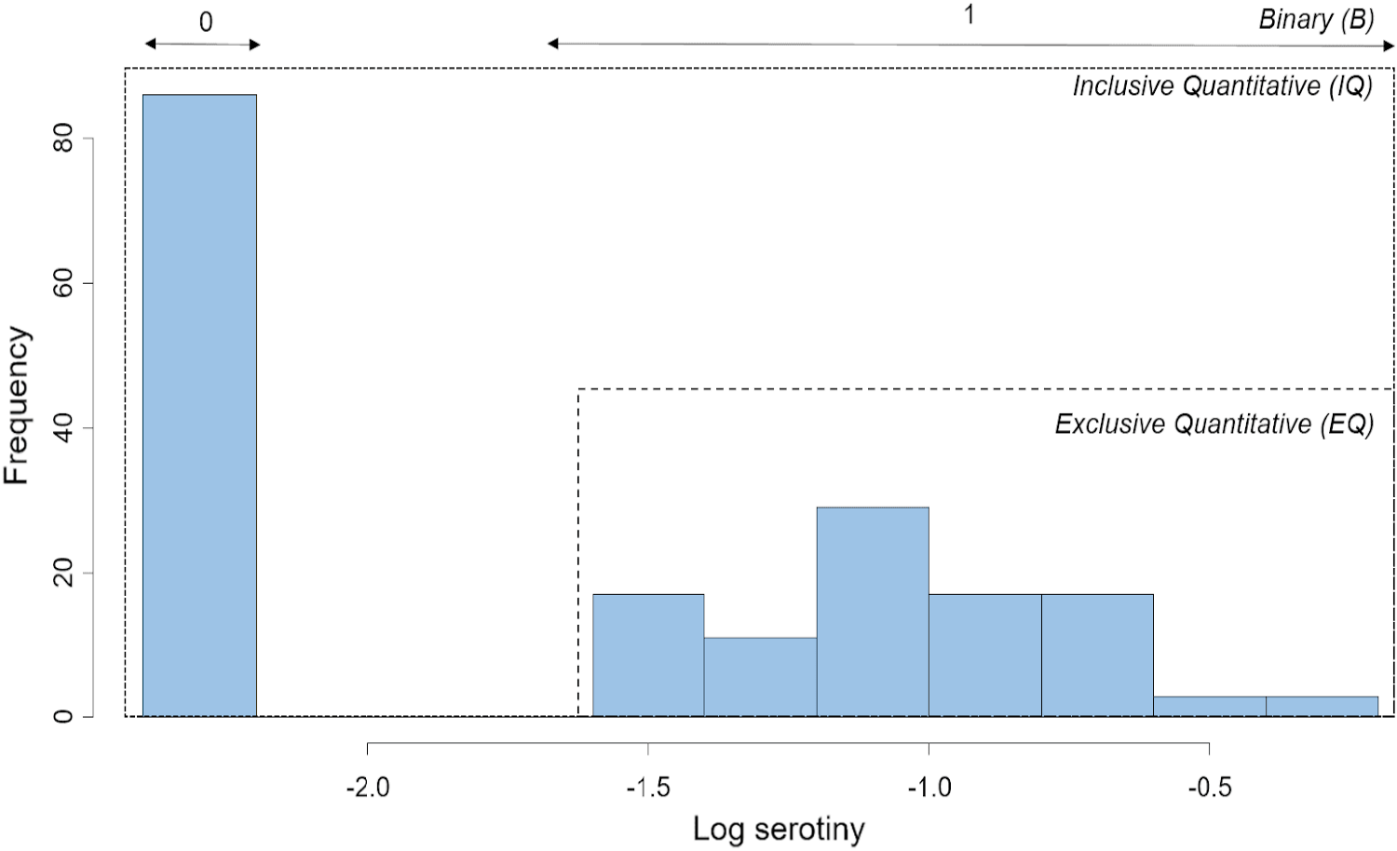
Histogram of distribution of the serotiny level (Log‐transformed) with the details of the three different types of data used for association analyses (“Binary” (B), “Exclusive Quantitative” (EQ), and “Inclusive Quantitative” (IQ)).

Because of the apparent bimodal distribution of trait values (see Results), serotiny data were transformed in three different ways (Figure 2):

- Serotiny level data were recorded as “0” for trees without serotinous cones, and as “1” otherwise; this allowed the analysis of the genetic bases of the presence/absence of serotiny. This is the *“Binary”* (“B”) data set.
- Serotiny level data were log‐transformed using the LogSt() function in the DescTools R package (Signorell, 2019), allowing for the Log‐transformation of zero values. This is the “*Inclusive Quantitative*” (“IQ”) data set.
- Trees with serotiny = 0 were removed, and then transformation proceeded as in (ii). This is the “*Exclusive Quantitative*” (“EQ”) data set.

The variation of serotiny level among populations and according to fire modality was assessed using an ANOVA, followed by a Fisher Least Significant Difference (LSD) test. As the data distribution was not normal, data were either log‐transformed or tested using the Kruskal–Wallis test followed by Bonferroni post hoc tests.

To remove the possible effect of some covariables on serotiny level, in order to keep only the genetic effect in the GWAS, residues of multivariate generalized linear models accounting for all environmental and ontogenetic variables (Table S1) were extracted in two ways:

- We designed a linear model of the form: TransformedSerotiny_x_ = μ + cofactor_1_ + cofactor_2_ + … cofactor_N_ + ε; the best model was identified using variable selection as implemented in the step() function of the R package “stats” (R Core Team, 2013) with AIC (Table S2) (“GLM” residues)
- all cofactors were used to perform a Principal Component Analysis using the FactoExtra R package (Kassambara & Mundt, 2017), and then, the first three components were used in the linear model: TransformedSerotiny_x_ = μ + PC_1_ + PC_2_ + PC_3_ + ε (“PCA” residues)

where TransformedSerotiny_x_ is one of the “Binary,” “Inclusive Quantitative,” “Exclusive Quantitative” data sets, and cofactor_i_ represents each cofactor listed in Table S1.

By combining the three data sets with our two types of linear models, we obtained six residue tables, named after the combination of data sets and linear models: “B‐GLM,” “IQ‐GLM,” “EQ‐GLM,” “B‐PCA,” “IQ‐PCA,” and “EQ‐PCA.” These six residue data sets were used in all association analyses; no other data set was used for this purpose.

### 2.3 Genotyping, background population structure, data filtering, and genotype imputation

Healthy and mature needles were sampled on the 192 trees chosen for serotiny genotype measurements and put in tagged individual paper bags. In the laboratory, samples were oven‐dried for 4 days at 30°C and then stocked with silica gel in order to avoid rehydration before DNA extraction. DNA isolation was performed using DNeasy® 96 Plant Kits from QIAGEN and genotyping was carried out at the Institute of Applied Genomics (IGA technologies services, Udine, Italy) based on 8000 SNPs, genotyped using the SPET technology (Scaglione et al., 2019). The target regions were chosen within coding sequences based on Pinosio et al. (2014).

Prior to analyses, the SNP data set was filtered as follows: (i) We removed variants with more than 20% missing data; (ii) we created subsets with the remaining variants so that no pair with linkage disequilibrium (LD) higher than 0.5 was left in each contig. To do this, we first computed LD using *vcftools v. 0.1.13* (Danecek et al., 2011), and then, we iteratively removed one variant from each pair of high LD variant pairs, until LD < 0.5 for all pairs, using an ad‐hoc R script.

Basic descriptors of the data set were obtained with *vcftools v. 0.1.13* (Danecek et al., 2011) and with the R package *“hierfstat” v. 0.5‐7* (Goudet, 2005). Genetic population structure was checked using (i) a principal component analysis (PCA) performed with the R package *“adegenet”* (Jombart, 2008; Jombart & Ahmed, 2011) and (ii) the Bayesian clustering method implemented in *fastSTRUCTURE 2.3.4* (Raj et al., 2014). K values ranging from 1 to 10 were implemented prior to obtaining values that explained population structure. Population pairwise divergence was estimated using Weir & Cockerham's *F*_ST_ (Weir & Cockerham, 1984) and the R package “*hierfstat” v. 0.5‐7* (Goudet, 2005).

Prior to genomic association analyses, we removed variants with a minor allele frequency (MAF) lower than 0.1 and we imputed missing genotypes using *Beagle 4.1* (Browning & Browning, 2016; Heer et al., 2018) without using a reference sequence. After the two filtering steps, 885 SNPs out of 8000 remained.

### 2.4 Relatedness matrix and heritability estimates

Patterns of relatedness must be checked and accounted for in the genomic association analyses. We obtained genetic relatedness matrices following three methods. First, Ritland's kinship (Ritland, 1996a) was computed from the SNP genotype data. Then, a genome‐wide relatedness matrix (GRM) was computed according to the GCTA (Yang et al., 2011) definition, as computed by the *snpgdsGRM()* function in the R package *“SNPrelate” v. 1.22.0* (Zheng et al., 2012) and finally as X_a_ × X_a_^*T*^, where X_a_ is the centered matrix of genotypes, as suggested by the manual of the R package *“mlmm.gwas”* (Bonnafous et al., 2018).

We used relatedness matrices and trait data to estimate heritability in two ways (see Table 1 for technical details):

- from Ritland's kinship and the value of the actual variance of kinship (Ritland, 1996b), as estimated in SPAGeDi (Hardy & Vekemans, 2002, as maintained at https://github.com/reedacartwright/spagedi), we computed Ritland's heritability (Ritland, 1996b). We used ad‐hoc R scripts to compute upper, central, and lower heritability estimates for serotiny (taken as residuals of multiple regressions, see above). Following this goal, first, the standard deviation of the numerator (covariance of trait‐based similarity and kinship) was obtained by bootstrap using the *bootstrap()* function in the R package *“bootstrap” v. 2019.6* (Tibshirani & Efron, 1993). Then, the standard deviation of the denominator (actual variance of relatedness) was obtained from SPAGeDi along with its central value (see above) and finally, the heritability, calculated from the ratio, was obtained from the central values (*m*_numerator_ *, m*_denominator_; central estimator) and from the pairs of values (*m*_numerator_ − sd_numerator_ *, m*_denominator_ + sd_denominator_; lower estimate) and (*m*_numerator_ + sd*m*_numerator_ *, m*_denominator_ − sd_denominator_; upper estimate).
- from the genome‐wide relatedness matrix (GRM) and traits (taken either as uncorrected data (data sets “B,” “IQ,” and “EQ”) or as residuals of multiple regressions, see above), Bayesian estimates of heritability (median and 95% credible intervals) from generalized linear mixed models were computed using the function *h2.jags()* of the R package *“gap” v. 1.2.2* (Zhao, 2008). When using uncorrected data, the same variables implemented as cofactors in the GLM were used as cofactors in heritability estimates.

**TABLE 1:** Heritability estimates obtained for the six data sets (B‐GLM; B‐PCA; IQ‐GLM; IQ‐PCA; EQ‐PCA, and EQ‐GLM), along with their lower and upper margins (Ritland's estimation) and confidence intervals (Bayesian estimation).

Data set	Lower margin	Central value	Upper margin	Lower credible interval	Central value	Upper credible interval
B‐GLM	0.0021	0.55	2.6	0.049	0.28	0.54
B‐PCA	0.037	0.79	3.5	0.18	0.45	0.67
IQ‐GLM	−0.16	0.70	3.9	0.17	0.43	0.70
IQ‐PCA	−0.15	0.84	4.8	0.27	0.56	0.80
EQ‐GLM	−0.45	−0.0046	1.7	0.00	0.15	0.64
EQ‐PCA	−0.44	0.090	2.0	0.00	0.11	0.57
B (GLM cofactors)		□	□	0.14	0.41	0.64
B (PCA cofactors)		□	□	0.0010	0.17	0.56
IQ (GLM cofactors)		□	□	0.0010	0.17	0.38
IQ (PCA cofactors)		□	□	0.0010	0.31	0.76
EQ (GLM cofactors)		□	□	0.0010	0.19	0.65
EQ (PCA cofactors)		□	□	0.0010	0.18	0.58

It is worth noting that heritability estimates were additionally provided by the MLMM approach used for GWAS (see below).

### 2.5 Genetic association analysis

The six data sets (“B‐GLM,” “IQ‐GLM,” “EQ‐GLM,” “B‐PCA,” “IQ‐PCA,” and “EQ‐PCA”) were used for running “random forest” analyses (machine learning algorithm; Breiman, 2001; Brieuc et al., 2018) to identify associations between SNPs and serotiny. The analyses were run with imputed missing data (see above), even if this process can induce a bias in the data (Heer et al., 2018). We carried out these analyses using two different R packages, *Boruta* (Kursa & Rudnicki, 2010) and *VSURF* (Genuer et al., 2015), for the six data sets described above, for a total of 12 association tests.

Association analyses were additionally carried out using the MLMM approach (Segura et al., 2012), which accounts for genetic relatedness, using the R package “mlmm.gwas” v. 1.0.6 (Bonnafous et al., 2018), with the integration of the additional mlmm_cof() function.

### 2.6 Functional annotation

Genes containing serotiny‐associated SNPs were annotated first by comparing them with *Arabidopsis thaliana*'s sequence database using BLAST (Basic Local Alignment Search Tool; Mount, 2007). Next, the best *A. thaliana* hits were annotated using the r package topGO (Alexa & Rahnenfuhrer, 2020), restricting the GO term search to the top hierarchical level (“biological process”). An enrichment test was performed with *p*‐values adjusted for multiple comparisons with the false discovery rate procedure (FDR) and a threshold of .05.

## 3 RESULTS

### 3.1 Phenotypic variation of serotiny level

**FIGURE 3:**
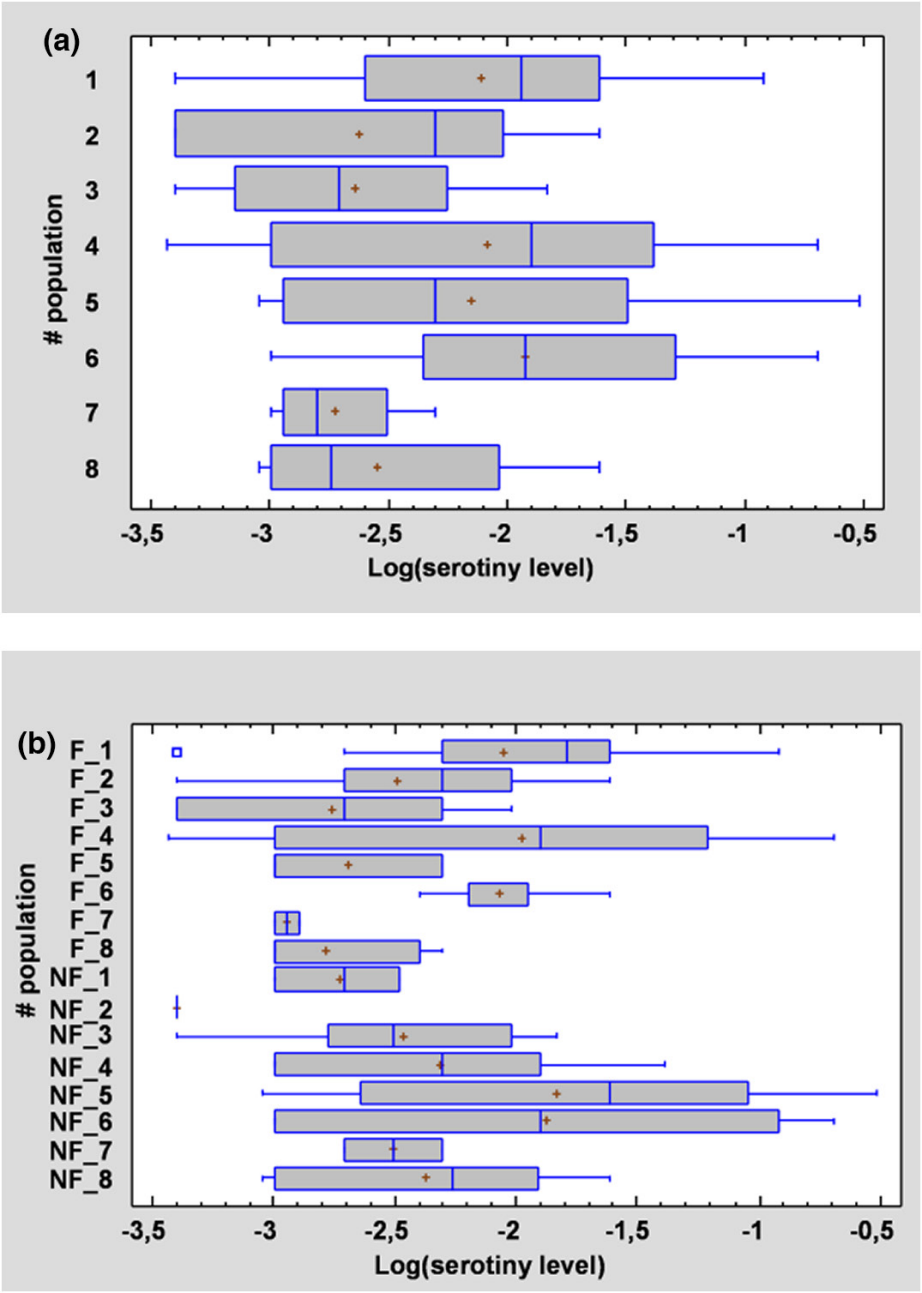
Variation of serotiny level among populations (a) and taking into account the fire modality (b) (F = Fire modality; NF = No‐Fire modality).

**FIGURE 4:**
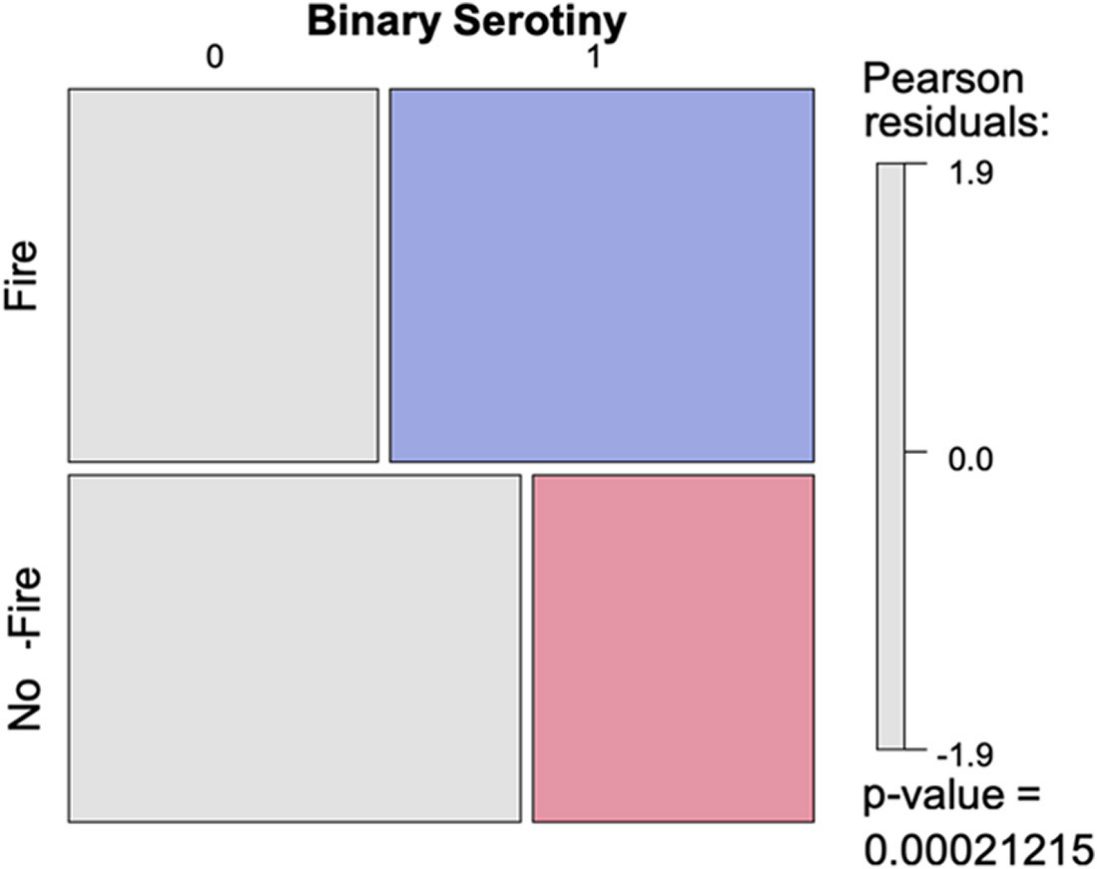
Mosaic of the binary serotiny difference between both fire modalities (“Fire” and “No‐Fire”) for trees expressing serotiny (1) or not (0). Fisher test *p*‐value <.001 (in blue: presence of serotiny in Fire populations; in pink: presence of serotiny in No‐Fire populations).

Serotiny level (quantitative data) ranged from 0 to 60% overall and was highly variable among populations regardless of the fire modality (Figure 3), for both raw (ANOVA, Kruskal–Wallis test, KW = 161.73, *p*‐value <.0001 with 33 significant comparisons according to the 95% Bonferroni test) and log‐transformed data (ANOVA, *F* = 3.49, *p*‐value <.0001 with 32 significant comparisons according to the Fisher Least Significant Difference (LSD) test). Taking into account only the data with serotiny level >0, the effect of the fire modality on the serotiny level was not significant (ANOVA, *F* = 0.84, *p* = .36). Furthermore, the number of trees expressing serotiny (i.e. with serotiny level > 0) was significantly higher in the “Fire” populations compared with the “No‐Fire” populations (Fisher test; *p*‐value = .0002; Figure 4). Data were discontinuously distributed, with a gap between trees displaying zero and nonzero serotiny (see Figure 2).

### 3.2 Genotype data and genetic structure of populations

Due to relatively high proportions of missing data and high levels of linkage disequilibrium among variants, we removed the vast majority of loci at the missing data and LD filtering steps. The final data set, prior to filtering for low‐frequency variants (MAF < 0.1), contained 904 biallelic SNP variants, and after the MAF filter, only 885 SNPs were left. While this reduced considerably genomic coverage, the remaining loci are fully independent and high‐quality, thus making the GWAS exercise more reliable, albeit very stringent. Average Nei's heterozygosity per SNP was 0.41 (standard deviation = 0.11) and there was a marked heterozygote excess (mean single‐locus *F*_IS_ within‐population: −0.44, standard deviation = 0.43).

**FIGURE 5:**
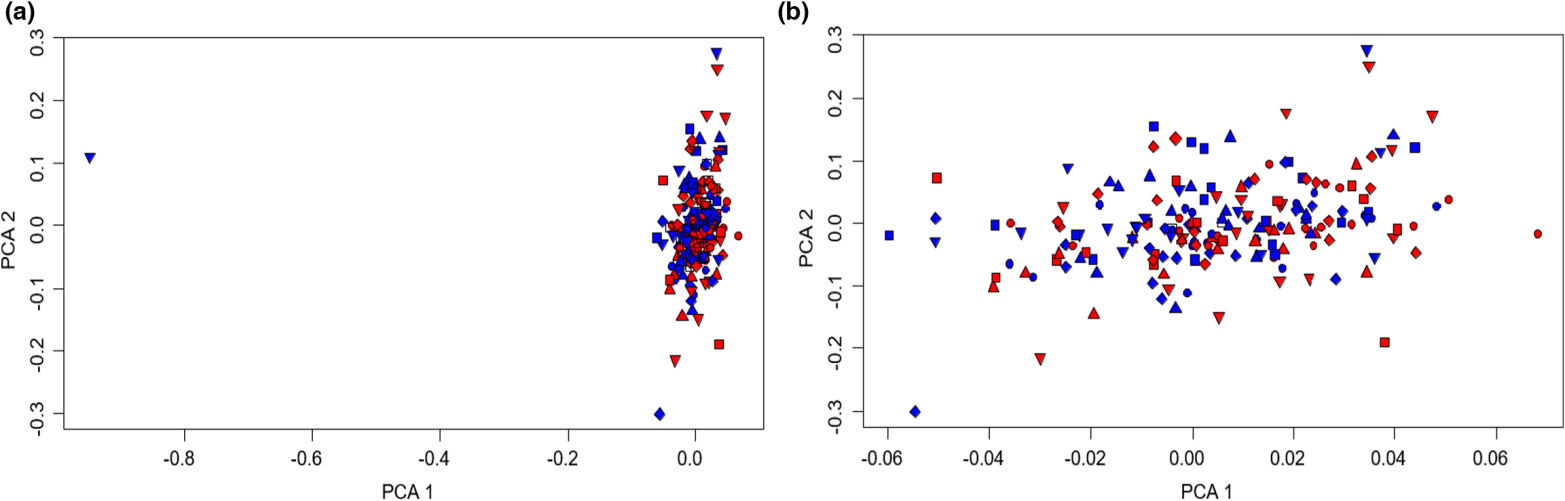
Principal component analysis biplots of the genetic structure of *Pinus halepensis* individuals according to the fire modality (a) data distribution on the two first components without removing the outlier, (b) data distribution after having removed the outlier (“Fire” populations in red and “No‐Fire” populations in blue).

Based on a principal component analysis, we chose to remove one outlier tree displaying a completely divergent genotypic structure (Figure 5a). After this step, the population structure was homogeneous, as indicated by the fact that the best number of clusters obtained with the Bayesian clustering analysis was *K* = 1. This suggests that there was not any confounding issue for association analyses, and therefore population structure was ignored in the subsequent analyses (Figure 5b). The estimation of pairwise population divergence (Weir and Cockerham *F*_ST_) also showed a very shallow structure, with very small and similar values in all pairs (Table 2).

**TABLE 2:** Pairwise genetic divergence (Weir and Cockerham *F*_ST_) between each *P. halepensis'* “Fire” (PHF) and “No‐Fire” (PNHF) populations.

	PHF_1	PHF_4	PHF_5	PHF_6	PHF_8	PHNF_1	PHNF_4	PHNF_5	PHNF_6
PHF_4	5.43E‐03								
PHF_5	4.44E‐03	4.49E‐04							
PHF_6	3.61E‐03	3.14E‐03	4.36E‐04						
PHF_8	5.48E‐03	1.65E‐03	2.34E‐03	1.55E‐03					
PHNF_1	1.51E‐03	5.48E‐03	3.66E‐03	4.38E‐03	4.95E‐03				
PHNF_4	7.35E‐03	3.84E‐03	1.65E‐03	3.40E‐03	6.42E‐03	4.45E‐03			
PHNF_5	7.44E‐03	3.10E‐03	1.38E‐03	4.08E‐03	3.85E‐03	4.70E‐03	1.06E‐03		
PHNF_6	6.48E‐03	6.52E‐03	7.29E‐04	3.98E‐03	5.73E‐03	8.88E‐03	4.83E‐03	5.47E‐03	
PHNF_8	5.87E‐03	3.12E‐03	−8.32E‐05	2.53E‐03	2.87E‐03	7.46E‐03	3.21E‐03	2.96E‐03	2.90E‐03

### 3.3 Relatedness and heritability

The distribution of the values of relatedness was skewed towards the right in all cases (D'Agostino skewness test, skew of 0.30, 4.2, and 4.4, respectively, for Ritland's, GCTA, and MLMM estimators; *p*‐value <10^−15^ in all cases), with a long, thin tail of excess values (Figure S1a). This suggests the presence of at least some small groups of tightly related individuals. Relatedness was not equally distributed among populations (Figure S1b); when comparing pairwise,” No‐Fire” numbers 5 and 6 (PHNF_5 and PHNF_6) had slightly higher relatedness than the matched fire‐prone stands (for GCTA estimates: *t*‐test, *t* = −2.2038, df = 319.78, *p*‐value = .02 for stand 5; *t* = −2.3775, df = 247.17, *p*‐value = .02 for stand; results are similar for the other estimators). The presence of unequally distributed relatedness values suggests that the kinship structure should be taken into account in GWAS analyses; however, the absolute numbers of pairs of individuals with very high kinship levels were small, suggesting that the bias may be relatively small. Heritability estimates were generally higher for the B and IQ type data sets and with the Ritland method, and lower in the EQ data set and with Bayesian estimations (Table 1). Central values varied between 0.28 and 0.84 for the G and IQ data sets but were much lowered when taking into account cofactors in the Bayesian analysis, down to 0.17–0.31 (with the exception of the B data set, for which introducing the cofactors increased the estimate). Estimates were close or equal to zero only for the EQ data set with the Ritland method (upper boundaries for the Ritland estimate went often beyond 1, while the Bayesian upper confidence interval limit was always lower than 1).

### 3.4 Identification of markers associated with serotiny level variation

SNPs identified by analyzing PCA‐based residuals (B‐PCA, EQ‐PCA, and IQ‐PCA) and GLM‐based residuals (B‐GLM, EQ‐GLM, and IQ‐GLM) only partially overlapped, regardless of the algorithm used (Table 3, “Union” column). The MLMM analysis did not return any significant result after the most stringent criteria were applied (Bonferroni‐corrected *p*‐value <.05 for parametric tests, extended BIC (e‐BIC) for likelihoods; Table S2).

**TABLE 3:** Number of SNPs found for each type of data used (*Quantitative 1: IQ*, *Quantitative 2: EQ*, and *Binary: B*), type of serotiny data correction (*GLM‐corrected* or *PCA‐corrected*), and type of random forest used (Boruta or VSURF, or union of PCA‐corrected and GLM‐corrected, or UNION taking into account all of combination between models and correction).

	GLM.corrected		PCA.corrected		Union	UNION
	Boruta	VSURF	Boruta	VSURF		
IQ	3	14	11	14	24	
B	7	12	6	16	22	40
EQ	5	11	6	10	15	

Globally, taking into account the number of SNPs in both types of correction (GLM‐ and PCA‐transformed), 24 significant SNPs in the *IQ* data set, and 15 in the *EQ* data set were associated with the variation of serotiny (both sets giving 37 different SNPs linked to the variation of serotiny). Twenty‐two SNPs in the *B* data set were associated with the presence/absence of serotiny. Only one SNP was significant in both *IQ* and *B* data set analyses (i.e., Contig2340_2217) and another one was significant in all the *EQ* data set analyses (i.e., clc_contig_12713_224).

Twenty genes containing significant SNPs were associated with biological processes having specific functions possibly involved in serotiny. They were divided into three classes: “Flowering,” “Stress tolerance,” and/or “Reproduction.” The list of loci driving the presence/absence of serotiny included genes involved in stress tolerance, while loci linked to the continuous variation of serotiny included flowering control genes (Table 4).

**TABLE 4:** Description of the SNPs associated with biological processes of interest.

SNP name	Model	Sequence	Gene name	Biological process
clc_contig_10191_90	EQ	AT5G01990	PILS6	Stress tolerance
clc_contig_1993_3310	EQ	AT2G06210	ELF8	Flowering
clc_contig_2167_2796	EQ	AT5G35750	HK2	Stress tolerance, Flowering
clc_contig_3226_4752	IQ, B	AT5G39710	EMB2745	Reproduction
clc_contig_5541_2946	IQ, B	AT1G55020	LOX1	Stress tolerance
clc_contig_6789_2014	B	AT5G13480	FY	Flowering, Reproduction
Contig1490_73	IQ	AT2G40890	CYP98A3	Stress tolerance
Contig2340_2217	IQ, B	AT4G34100	CER9	Stress tolerance
Contig2371_653	EQ	AT1G01040	DCL1	Flowering, Reproduction
Contig5893_417	IQ, B	AT1G33410	SAR1	Flowering
Contig6652_237	B	AT2G46660	CYP78A6	Reproduction (seed)
Contig6777_720	EQ	AT4G18780	IRX1	Stress tolerance
Phal_PtaS20492475_294	EQ	AT1G28520	VOZ1	Stress tolerance, Flowering
Phal_PtaS25086892_281	B	AT1G71860	PTP1	Stress tolerance
clc_contig_161_4027	EQ	AT5G49160	MET1	Flowering
clc_contig_2079_4430	IQ	AT4G29380	VPS15	Pollen germination
clc_contig_4658_2921	B	AT5G62790	DXR	Stress tolerance
clc_contig_9287_2067	EQ	AT5G05350	PLAC8	Stress tolerance
Contig1655_1637	B	AT3G43300	ATMIN7	Stress tolerance
Contig1165_2751	IQ	AT1G55250	HUB2	Flowering, Reproduction

## 4 DISCUSSION

Our study provides new important information on the genetic bases of serotiny, one of the most studied fire‐related traits in fire‐embracer species such as *Pinus halepensis*. Using an in situ genomic association study, we identified several SNPs and a variety of candidate genes that could underlie fire phenotypes associated with the variation of serotiny level observed in the field, as loci associated with serotiny might be associated with other correlated fire adaptive traits. However, our results differed from previous ones from the point of view of population structure. Indeed, population kinship structure can be affected by the population dynamics driven by fire events (Budde et al., 2017), with potentially closer kinship in fire‐prone populations. Nevertheless, we did not detect such patterns in our populations. This could be due to a difference in fire intensity and frequency (fire regime), our study areas being less affected by fires than the ones of Budde et al. (2017), for instance. At the population level, the small levels of divergence are in agreement with previous studies using similar approaches (Scotti et al., 2023).

Previous studies on serotiny suggested that, because trees of different pine species could be serotinous or not, this trait was driven by a simple genetic control involving only one gene (Givnish, 1981; Talluto & Benkman, 2013; Teich, 1970). More recently, serotiny was recognized as a continuous trait, driven by numerous genes involved in several functions (Budde et al., 2014; Hernández‐Serrano et al., 2014; Lamont et al., 2020). In our study, the overall distribution of serotiny level was bimodal, with a large peak at zero and a long, flat continuous tail between 3% and 60% and a mean serotiny level (13 ± 11%) much lower than in other studies (e.g., Castellanos et al., 2015). Serotiny level can be driven by many factors, such as environmental conditions or tree age (i.e. ontogeny) and, of course, by fire frequency (Martín‐Sanz et al., 2017; Ne'eman et al., 2004; Romero & Ganteaume, 2020). As fire frequency is lower in southeastern France than in some regions of Spain or Portugal (JRC PESETA IV, 2020), this could explain this difference in serotiny level. We also observed high variability in serotiny level among trees, agreeing with previous studies (Hernández‐Serrano et al., 2013; Tapias et al., 2004).

“Fire” and “No‐Fire” populations significantly differed regarding the presence/absence of serotiny but did not show any difference in the distribution of nonzero (quantitative) values of serotiny. This could be explained by the small difference between our fire modalities (i.e. 0, 1, or 2 fires), and by the fact that fire events have affected the populations only over the last few generations. Indeed, given the small number of generations since the start of the selection and the weak difference between fire regimes, selection could have had a stronger effect on population divergence at a threshold trait (such as presence/absence of serotiny) than at a continuous trait (such as level of serotiny).

Forty SNPs were associated with serotiny, out of which 22 were associated with a binary component of serotiny variation (that is, with the trait being treated as 0 = no serotinous cones, 1 = fraction of serotinous cones above X%; remind that no tree had a proportion of serotinous cones between 0 and X%). While it seems counter‐intuitive that a binary component of a trait has a polygenic control, this could be explained by the fact that the action of multiple genes is needed to trigger the development of serotiny, perhaps in a redundant way and through threshold effects. Notice, though, that serotiny is a complex, derived trait that may be driven by many other parameters, themselves under polygenic control, which may explain this result. Six significant genes for binary variation (*B* and *IQ* models) were associated with biotic and/or abiotic stress tolerance, such as temperature, drought, light, or pest attack, which were already identified in several previous studies (Brown et al., 2005; Chen et al., 2005; Feraru et al., 2019; Lü et al., 2012; Song et al., 2018; Xing et al., 2010). Budde et al. (2014) found eight loci in *P. pinaster* associated with serotiny level variation, also involved in stress tolerance linked to water deprivation or high temperatures. Three significant loci lie in genes controlling seed development, end of the dormancy, and embryo sac development (EMB2745, HUB2, and CYP78A6). Seeds from serotinous cones have indeed been shown to differ from those of nonserotinous cones for insulation against high temperatures, which stimulates their germination compared with those in nonserotinous cones and is linked to a different enzymatic activity (Goubitz et al., 2002, 2004; Moya et al., 2013). However, no indication of gene association between herbivory resistance and serotiny emerged from our GO term analysis. Indeed, granivores can select for low serotiny, since serotiny exposes seeds longer to a risk of consumption (by squirrels that are quite frequent in the study area, for instance).

Quantitative variation in serotiny levels (i.e. *EQ* models) was also controlled by loci determining responses to water deprivation or high temperature (PILS6, IRX1, HK2, RBP‐DR1) but was also driven by genes controlling flowering (i.e. ELF8, HK2, MET1, DCL1, and VOZ1), already identified in previous studies (Celesnik et al., 2013; Shiraya et al., 2008; Tsuzuki et al., 2014). ELF8 (Early flowering 8), DCL1 (Dicer‐Like 1), MET1, and VOZ1 (Vascular plant One‐Zinc finger) control flowering time (Kankel et al., 2003; Tsuzuki et al., 2014; Yu & Michaels, 2010). Their involvement in the determination of serotiny level suggests that developmental processes of reproductive organs are key components of the trait.

Contig2340_2217 was the only significant locus shared by the IQ and B models. This SNP is located in gene CER9, which could be involved in several important functions for the serotinous cone formation. One of these functions is cuticle development, affecting seed permeability (Sieber et al., 2000), water loss through stomata (Lu et al., 2012), resistance to pathogen attack, plant thermo‐tolerance (Li et al., 2017), and flower opening (Chen et al., 2019). Other important functions are wax production (Rashotte et al., 2001), playing a role in seed thermic insulation and water permeability in serotinous cones (Keeley et al., 2011; Moya et al., 2008; Salvatore et al., 2010) and suberin production (Lee & Suh, 2015), also affecting thermal insulation of serotinous cones and seeds. Finally, biosynthesis of isoprenoids (also controlled by gene DXR, identified in *B* models), which are easily flammable compounds, and the building blocks of terpenes occurring in resin are also involved in serotinous cone formation.

The MLMM approach did not return any significant result. This may be due to differences in precision or stringency between methods, as also suggested by the smaller estimation of background genome‐wide heritability obtained with MLMM than with other methods, also controlling for relatedness (Table 1, vs Table 2, Bayesian estimates). As a matter of fact, it is hard to say whether, conversely, the methods that have detected more signals are too liberal; it is well known that, with relatively small populations, allelic effects can be overestimated (Xu, 2003).

While our study has returned significant associations between sequence variants and serotiny, the genomic coverage of our SNP set is quite small. It represents a few percent of gene space—assuming that we represent slightly fewer than 1000 genes and that there are 20,000–30,000 unique genes in a pine genome—and a very small fraction of the very large pine genome. The fact that even just a handful of positive signals could be found with such a narrow sample of the potential variant space suggests that many genomic regions may control the trait.

### CONCLUSIONS

We observed a higher proportion of serotinous trees in the “Fire” than in the “No‐Fire” populations, notwithstanding the fact that populations have undergone only a few fire events over the last six decades. This suggests that population levels of serotiny can quickly respond to a change in fire frequency, in agreement with previous studies (Talluto & Benkman, 2013). This result confirms the strong relationship between fire and *P. halepensis*, and the ability of this species to grow under recurrent fire events with this key functional trait selected over generations.

Our heritability estimates confirm that serotiny is a moderately to highly heritable trait for *P. halepensis*. Our association analyses returned 40 genes associated with serotiny, approximately half for its presence/absence component and half for its quantitative component, supporting a polygenic control of the trait, contrary to what was suggested in several studies (Givnish, 1981; Talluto & Benkman, 2013; Teich, 1970) but agreeing with the conclusions of Budde et al. (2014) and Lamont et al. (2020). The observed bimodal trait distribution suggests separate genetic bases for the presence/absence of serotinous cones and serotiny level. The variety of gene functions controlling serotiny suggests that adaptation to fire‐prone environments through serotiny involves chemical properties (terpene levels), developmental cues (development of reproductive organs), and stress resistance. This stresses the high potential of *P. halepensis* to adapt to fire through its fire‐embracer strategy, benefiting from a large and flexible genetic basis of trait variation.

The ongoing climate crisis will increase fire risks and fire frequency in the Mediterranean basin (Dupuy et al., 2020). *P. halepensis* seems to be a good candidate to adapt to this change. Yet, if fires become too recurrent, then other components of serotiny as a trait, namely age to maturity and age of the first serotinous cone production, will become crucial, and will need to be closely scrutinized, to more precisely assess the species' promising resilience level.

## AUTHOR CONTRIBUTIONS

**Bastien Romero:** Conceptualization (supporting); data curation (lead); formal analysis (equal); investigation (lead); methodology (supporting); visualization (equal); writing – original draft (lead); writing – review and editing (equal). **Ivan Scotti:** Formal analysis (lead); funding acquisition (equal); investigation (equal); methodology (equal); supervision (equal); validation (lead); visualization (equal); writing – original draft (equal); writing – review and editing (lead). **Bruno Fady:** Conceptualization (equal); funding acquisition (supporting); investigation (equal); supervision (equal); validation (lead); writing – original draft (supporting); writing – review and editing (supporting). **Anne Ganteaume:** Conceptualization (equal); data curation (supporting); funding acquisition (equal); investigation (supporting); project administration (lead); resources (supporting); supervision (equal); validation (supporting); visualization (supporting); writing – original draft (supporting); writing – review and editing (equal).

## CONFLICT OF INTEREST STATEMENT

The authors know of no conflicts of interest associated with this publication.

## Supporting information


Appendix S1

## Data Availability

Single Primer Enrichment Technology genotypes are available at https://data.inrae.fr/dataset.xhtml?persistentId=doi:10.15454/7COC1A. Data on serotiny is accessible at the Dryad Digital Repository: https://doi.org/10.5061/dryad.280gb5mth.
