# Supplementary material for: Fire frequency, as well as stress response and developmental gene control serotiny level variation in a widespread pioneer Mediterranean conifer, Pinus halepensis
Source: Ecol Evol. 2023 Mar 21;13(3):e9919. doi: 10.1002/ece3.9919 (PMC10030233; doi:10.1002/ece3.9919)
Supplement: Supplementary file 1 — Appendix S1 [file ECE3-13-e9919-s001.docx]

## Supplementary information

**Table S1** Cofactors used to corrected serotiny data for each “Fire” (PHF) and “No-Fire” (PHNF) *Pinus halepensis* ‘populations for the GWAS analyses.

| Population | Fire modality | Elevation (m) | Basal area (m^2^ ha^-1^) | Slope (%) | Exposure (°) | Age (years) | Latitude (°) |
| --- | --- | --- | --- | --- | --- | --- | --- |
| PHF_1 | Fire | 600 | 14.00 | 30 | 182 | 21.17 (2.04) | 44.267 |
| PHNF_1 | No-Fire | 590 | 19.20 | 20 | 210 | 26.10 (2.06) | 44.254 |
| PHF_4 | Fire | 340 | 6.38 | 25 | 175 | 19.15 (2.13) | 43.676 |
| PHNF_4 | No-Fire | 180 | 7.33 | 27 | 180 | 20.82 (2.32) | 43.696 |
| PHF_5 | Fire | 217 | 7.50 | 20 | 188 | 20.16 (3.48) | 43.499 |
| PHNF_5 | No-Fire | 240 | 20.00 | 10 | 182 | 21.10 (2.00) | 43.491 |
| PHF_6 | Fire | 95 | 27.00 | 12 | 161 | 22.95 (2.04) | 43.390 |
| PHNF_6 | No-Fire | 160 | 27.67 | 25 | 180 | 22.89 (2.30) | 43.460 |
| PHF_8 | Fire | 150 | 14.00 | 20 | 140 | 24.10 (2.15) | 43.190 |
| PHNF_8 | No-Fire | 170 | 15.25 | 40 | 137 | 21.33 (4.39) | 43.234 |

**Table S2.** Background genomic heritability estimates obtained using MLMM method. Data set: combination of type of data transformation/subsetting and of type of cofactors used in the analysis (see Material and Methods); residuals: analyses performed on the residuals of multiple regressions against the cofactors; cofactors: analyses performed by including the cofactors in the model.

| Data sets | Residuals | Cofactors |
| --- | --- | --- |
| B / GLM | 0.0 | 0.084 |
| B /PCA | 0.0 | 0.11 |
| IQ / GLM | 0.028 | 0.0 |
| IQ /PCA | 0.0 | 0.0 |
| EQ / GLM | 0.024 | 0.016 |
| EQ / PCA | 0.081 | 0.0 |


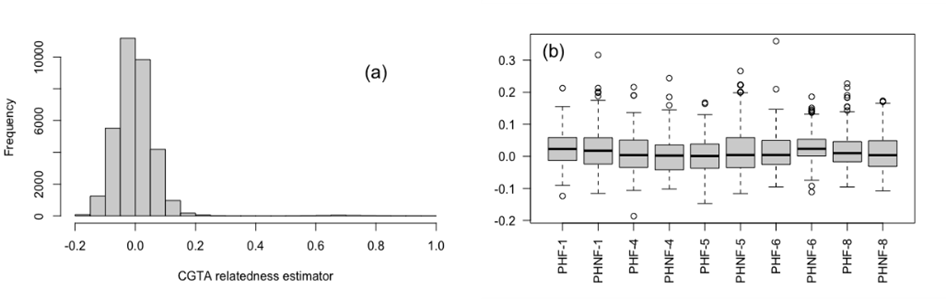


Figure S1. Distribution of the values of GCTA estimates of genetic relatedness (GRM, genome-wide relatedness matrix). (a) Histogram of the values for all populations; (b) box-plot of values by population.
